# Shedding Light on the Role of Extracellular Vesicles in HIV Infection and Wound Healing

**DOI:** 10.3390/v12060584

**Published:** 2020-05-27

**Authors:** Aseel Alqatawni, Adhikarimayum Lakhikumar Sharma, Beatrice Attilus, Mudit Tyagi, Rene Daniel

**Affiliations:** 1 Center for Translational Medicine, Thomas Jefferson University, 1020 Locust Street, Philadelphia, PA 19107, USA; aseeltq@yahoo.com (A.A.); LakhikumarSharma.Adhikarimayum@jefferson.edu (A.L.S.); Beatrice.Attilus@jefferson.edu (B.A.); mudit.tyagi@jefferson.edu (M.T.); 2 Farber Hospitalist Service, Department of Neurological Surgery, Thomas Jefferson University, Philadelphia, PA 19107, USA

**Keywords:** extracellular vesicles, exosomes, HIV, AIDS, wound healing, immune response

## Abstract

Extracellular vesicles (EVs) play an important role in intercellular communication. They are naturally released from cells into the extracellular environment. Based on their biogenesis, release pathways, size, content, and function, EVs are classified into exosomes, microvesicles (MVs), and apoptotic bodies (ApoBDs). Previous research has documented that EVs, specifically exosomes and MVs, play an important role in HIV infection, either by promoting HIV infection and pathogenesis or by inhibiting HIV-1 to a certain extent. We have also previously reported that EVs (particularly exosomes) from vaginal fluids inhibit HIV at the post-entry step (i.e., reverse transcription, integration). Besides the role that EVs play in HIV, they are also known to regulate the process of wound healing by regulating both the immune and inflammatory responses. It is noted that during the advanced stages of HIV infection, patients are at greater risk of wound-healing and wound-related complications. Despite ongoing research, the data on the actual effects of EVs in HIV infection and wound healing are still premature. This review aimed to update the current knowledge about the roles of EVs in regulating HIV pathogenesis and wound healing. Additionally, we highlighted several avenues of EV involvement in the process of wound healing, including coagulation, inflammation, proliferation, and extracellular matrix remodeling. Understanding the role of EVs in HIV infection and wound healing could significantly contribute to the development of new and potent antiviral therapeutic strategies and approaches to resolve impaired wounds in HIV patients.

## 1. Introduction

The prevalence of HIV/AIDS has expanded and spread across the globe since its first detection in the early 1980s [1,2]. The inability to find a cure made HIV one of the most dreaded pathogens ever known. However, the introduction of highly active antiretroviral therapy (HAART) immensely reduced the morbidity and mortality rate among HIV-infected individuals [3,4]. Continuous use of HAART inhibits viral replication, controls new infections, and increases life expectancy [5]. Even though the current HAART treatment regimens have greatly improved the life expectancy of HIV/AIDS patients, they fail to eliminate the virus from the body completely, and HIV persists in cellular reservoirs because of latency establishment, cryptic ongoing replication, and poor drug penetration [6]. 

HIV replication weakens the immune system, reducing the ability to fight back against invading foreign pathogens. Consequently, following immunodeficiency, HIV-infected individuals succumb to common infections, such as tuberculosis (TB), hepatitis C virus (HCV), and other opportunistic infections. At advanced stages of HIV infection, patients are also at risk of wound-healing complications [7,8] and other wound-related issues. Moreover, HIV patients experience an increased incidence of perioperative complications, such as infection, poor healing, and mortality [9,10,11]. Many perceive that any kind of surgery poses greater risks to HIV-infected individuals than to uninfected individuals due to their susceptibility to super-infections and poor wound healing.

Recently, research has shown that extracellular vesicles (EVs) play an important role in HIV replication and its associated complications. EVs are small plasma-membrane-derived particles that carry a complex cargo of nucleic acids, lipids, and proteins [12,13,14,15,16] and are known to have a variety of important physiological effects [17]. Almost all cell types secrete EVs into the extracellular environment [18,19]. Vesicles secreted from platelets, leukocytes, and endothelial cells are known to play a crucial role in activating several fundamental cells, including vascular smooth muscle cells. The intrinsic activity and immunomodulatory properties of EVs contribute to regulating vascular inflammation, tissue regeneration, and vascular repair. Studies have shown that EVs may be involved in wound healing by controlling cellular processes, including cell proliferation and migration in ways that accelerate the wound-healing process [20,21,22]. EVs have been shown to play a role in a variety of viral infections, with EVs released from the infected cells influencing the spread of viruses. For example, the Epstein–Barr virus (EBV), which can cause tumors in humans, uses EVs to transfer viral oncoprotein, latent membrane protein 1 (LMP-1), and virus-encoded miRNAs to normal cells. EVs released from EBV-infected cells show the presence of latent-phase viral proteins LMP2, Epstein–Barr nuclear antigen 1 (EBNA1), and EBNA2 [23]. EVs released from Coxsackievirus-B1-infected cells can spread the virus to the secondary site [24]. EVs are also considered carriers for Flavivirus transmission from arthropod to human cells [25]. HIV has also been shown to alter EV content and utilize the EV-secretion pathways to modulate its pathogenesis. Recently, research on the role of EVs in HIV in particular has been expanding at a rapid pace. This review aimed to update the current knowledge about the roles of EVs in HIV infection and wound healing. We have detailed the underlying molecular mechanisms that govern wound healing and how exosomes contribute to wound healing. Subsequently, we have described the impact of HIV infection on perturbing both exosomal and wound-healing pathways.

## 2. Extracellular Vesicles and Their Types

EVs are membrane-enclosed vesicles that are naturally released from cells as part of their normal physiology and during acquired abnormalities. They play an important role in intracellular and intercellular communication [18,19,26], including regulating the immune response, cell proliferation, cell migration, blood vessel tube formation, and cancer progression, among other biological processes [27]. The transport and transfer of EVs influence various physiological and sometimes pathological functions within their target cells [27]. The significance of EVs depends on the delivery of their contents to recipient cells, thereby altering the cellular and biological process [27]. EV contents vary depending on the generating cells, from which they acquire lipids, nucleic acids, and proteins [28,29]. Based upon their biogenesis, release pathways, size, content, and function, EVs may be broadly classified into exosomes, microvesicles (MVs), and apoptotic bodies [17,27,30,31,32].

### 2.1. Exosomes

Exosomes are a type of EV generated by inward budding of the limited multivesicular body (MVB) membrane. Even though this mechanism of exosome formation is poorly understood, it is widely accepted that exosomes are formed and developed via the endocytic pathway [33]. Invagination of late-endosomal membranes leads to the formation of intraluminal vesicles (ILVs) within large MVBs. These ILVs are then released into the extracellular environment upon fusion with the plasma membrane. These are referred to as exosomes and are typically 30–100 nm (average ~100 nm) in diameter [26,28,29,34]. They contain RNAs, including messenger RNAs (mRNAs) and microRNAs (miRNAs), lipids (cholesterol, sphingomyelin, ceramide, phospholipids, and glucans), and protein from the cells [35]. Exosomes are taken up by distant cells, where they influence the function and behavior of the cells. The content of exosomes (e.g., nucleic acids, protein, metabolites) affects the biological responses of recipient cells. Exosomes are believed to be involved in removing excess and/or unnecessary constituents from cells to maintain cellular homeostasis. They are also associated with immune responses, cardiovascular diseases, viral pathogenicity, central-nervous-system-related diseases, and progression of cancer. Exosomes are secreted from a variety of cells and may either promote or restrict the development of disease [35]. Exosomes could potentially be used as biomarkers of infectious diseases and for preventing infections. The intrinsic properties of exosomes have advanced their potential use in the therapeutic control of many diseases [35]. 

### 2.2. Microvesicles (MVs)

MVs are a type of EV that form by direct outward budding, or pinching, of the cells. The formation and shedding of MVs from the cell surface are not yet fully understood. It is hypothesized that MVs are formed because of the interplay between phospholipid redistribution and cytoskeletal protein contraction [36,37]. MVs range in size from 100 nm to 1 µm in diameter [26,28,29,34,38]. They mainly contain highly concentrated plasma- and cytosol-associated proteins [39,40], including cytoskeletal proteins, heat-shock proteins, integrins, and proteins containing post-translational modifications such as glycosylation and phosphorylation [41,42]. They are involved in cell–cell communication between local and distant cells and are highly similar to exosomes in clinical settings. Therefore, they can also be engineered to deliver therapeutic elements, including short interfering RNAs, antisense oligonucleotides, chemotherapeutic agents, and immune modulators, to desired targets [35].

### 2.3. Apoptotic Bodies (ApoBDs)

Apoptotic cells release EVs that are known as apoptotic bodies (ApoBDs) into extracellular environments. ApoBDs are formed via separation from the cytoskeleton as a result of increased hydrostatic pressure after the cell contracts. ApoBDs are released into extracellular spaces via several stages: cell membrane contraction, condensation of cytoplasm, shrinkage of cell size, alteration and condensation of nuclear chromatin, and then deterioration of plasma membrane. Finally, the plasma membrane undergoes blebbing and the cellular contents are disintegrated into distinct membrane-enclosed vesicles. These membrane-enclosed vesicles are known as apoptotic bodies and their sizes range from 50 nm up to 5000 nm in diameter [26,43]. They contain intact organelles, chromatin, and small amounts of glycosylated proteins [26,44,45,46] which help to remove dying cells [47]. They also serve as key regulators of antigen presentation [47], and therefore they are also becoming a key player in immune modulation.

## 3. Extracellular Vesicles (Particularly Exosomes) Versus HIV Virion Characteristics

**Figure 1:**
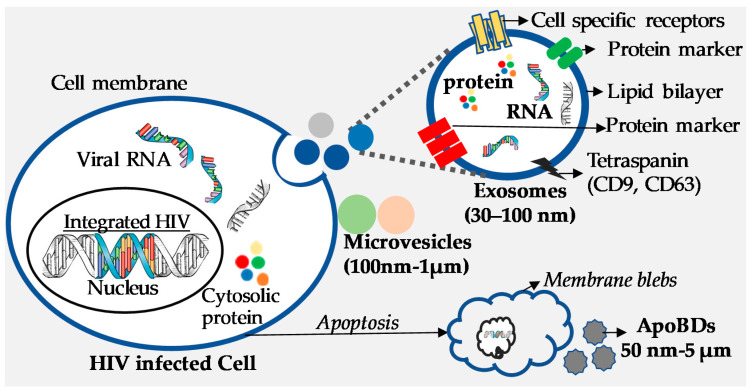
Extracellular vesicles (exosomes, microvesicles, and apoptotic bodies) secreted from HIV-infected cells. Exosomes (30 nm–100 nm) contain proteins, nucleic acids (RNA), and lipids.

In the past decade, EVs have been shown to influence numerous cellular functions. Infected cells secrete EVs just as normal cells do, and during pathogenic infection, EVs secreted from infected cells carry not only host components but also pathogen-derived components [48,49]. Consequently, EVs produced from infected cells modulate the susceptibility of the receiving cell, usually by priming the cell for infection [50]. Similarly, in HIV infection, infected cells shed not only HIV virions, but also release EVs which, besides containing host-cell components also carry HIV components (Figure 1). Exosomes, the predominant population of EVs, resemble HIV virions in terms of their biogenesis, physical properties, size, and density (ranging from 1.13–1.21 g/mL) [44]. Both are surrounded by a phospholipid bilayer and possess a similar composition, including lipids [51], carbohydrates [52], proteins [53,54], and RNA [55]. Exosomes derived from HIV-infected cells are enriched with viral proteins such as Nef and viral RNAs. Because of these similarities, HIV virions are believed to be generated from the same pathway as the exosomes [56,57]. Size-wise differentiation of HIV and exosomes is quite difficult, which poses a challenge when isolating exosomes in pure form, as well as when characterizing their precise role and contribution to disease pathogenesis in HIV-infected individuals. 

**Figure 2:**
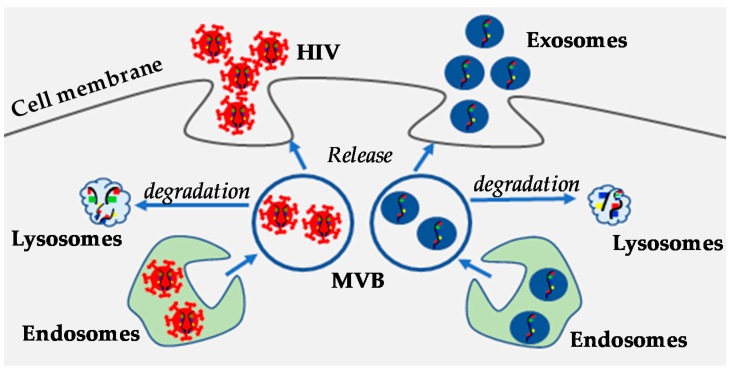
The exosome/microvesicle biogenesis pathway mediates HIV budding. HIV budding (red color) and exosome release (blue color) share similar pathways.

Although exosomes and HIV virions share biochemical features (Figure 2), there are certain differences between these two. HIV virions are more organized and uniform in structure regardless of the source of infected cells. On the other hand, the structure of exosomal vesicles varies depending on the parental cell. The biochemical content of HIV virions is more or less consistent, while the content of exosomes is highly diverse depending on the source [58]. Another important difference is that HIV virions replicate, while exosomes do not replicate. Although exosomes may contain virus-associated nucleic acids and proteins, true exosomes are metabolically inert. Therefore, they are unable to replicate their content and cannot generate progeny. 

## 4. Extracellular Vesicles Modulate HIV Infection

Cells infected with microorganisms, including bacteria, fungi, and viruses, produce EVs that can either modulate host immunity or enhance the severity of the infection [48,49]. The EVs produced by infected or sick cells commonly incorporate virulent factors, and, as a result, these EVs play a crucial role in spreading the infection or sickness [59,60]. It has been noted that EVs released from infected cells can mediate the inhibition of immune responses mainly by accelerating the apoptosis of immune cells [59]. Studies have clearly shown the role of EVs in shielding certain pathogens from the host immune system, and thus promoting the systemic spread of infection. On the other hand, EVs, especially from uninfected/healthy cells, have been found to act against infections by restraining the proliferation and transmission of pathogens, mainly viruses [50,59,61]. Like any other pathogenic infection, HIV-infected cells also secrete EVs that modulate HIV pathogenesis. In most cases, but not all, exosomes secreted from HIV-infected cells enhance HIV infection and disease progression. However, exosomes from uninfected cells usually carry HIV-inhibitory and -protective properties. Overall, evidence suggests that the exosomal effect on HIV mainly depends on their cellular origin [62]. The role of exosomes in HIV infection/transmission/disease progression, based on their origin, is discussed here. 

### 4.1. Blood/Serum/Plasma

Human blood is the main biofluid responsible for HIV transmission. The blood contains different types of cells, a fraction of which are HIV-susceptible. All these cells secrete exosomes. Exosomes released from HIV-1-infected cells have distinct constituents, and they are thus functionally different from the exosomes released by uninfected cells. Analysis has revealed that the number of exosomes secreted [63] and the levels of cytokines and chemokines [64] in exosomes are significantly higher in plasma samples from HIV-1-infected individuals than uninfected individuals. This correlates well with HIV disease progression [63,64]. HIV-infected cells also secrete exosomes that contain chemokine receptors, CCR5, and CXCR4. These receptors are delivered to the recipient or uninfected cells to facilitate HIV establishment and spread in CD4+ cells. EVs carrying CCR5+ microparticles, released by CCR5+ peripheral blood mononuclear cells (PBMCs) [65], transfer CCR5 coreceptor to CCR5-CD4+ cells, which allows infection of CCR5-CD4+ cells with R5-tropic HIV-1 [65]. Likewise, the microvesicle-mediated transfer of CXCR4 coreceptor to the recipient cells enhances the infection with X4-tropic HIV-1 [66]. Hence, besides priming the uninfected cells, exosomes may facilitate the cellular entry of restricted HIV strains, thus modifying viral tropism [67]. Exosomes from HIV-infected cells contain Nef protein which, in turn, enhances the exosomes’ secretion [68]. Nef-containing exosomes can induce CD4+T-cell apoptosis in vitro, defining the role of exosomes in the T-cell depletion [68]. Nef protein promotes infection by activating uninfected cells. During exosome biogenesis, HIV also incorporates transactivating response (TAR) RNA into exosomes [69,70]. Therefore, the exosomes thus produced from the infected cells contain viral protein, and TAR supports HIV infection in recipient cells [70,71,72]. Researchers have found that TAR-RNA-element-harboring exosomes stimulate proliferation, migration, and invasion of transformed cells, and TAR-carrying EVs promote proliferation by lowering the level of pro-apoptotic protein and enhancing the expression of proto-oncogenes [71]. Moreover, TAR also enhances HIV replication by inhibiting the interferon-induced protein kinase PKR and increasing the translation of viral mRNA [72]. A study by Sampey et al. indicated that exosomes that contain TAR RNA induce secretion of pro-inflammatory cytokines, specifically TNF-β and IL-6, from monocyte-derived macrophages (MDM) [69]. Researchers also found that infected T cells released exosomes containing active ADAM metallopeptidase domain 17, which induced activation and replication of HIV [73]. Thus, by priming the uninfected cells for infection, exosomes contribute to viral transmission and persistence [49]. Interestingly, TAR is also present in the exosomes isolated from the serum of HAART-treated HIV patients, validating that even with HAART treatment, HIV transcription is still going on [74]. Exosomes secreted from HIV-infected cells modulate matrix deposition and vascular permeability through communication with fibroblasts and endothelial cells [75]. This process further contributes to the spread of HIV and other coinfections [76]. The transportation of HIV proteins and RNA by exosomes has also been implicated in chronic inflammation, leakiness of gut or blood–brain barrier endothelium lining, and long-term neurological dysfunction. EVs derived from HIV-1 were also found to suppress host immune responses and to enhance viral expansion depending on the target cell [49]. 

Most exosomes released by infected cells have stimulatory effects on HIV-1 infection; however, a certain population of exosomes has an inhibitory effect on the HIV life cycle, including transcription [30,49,77,78]. They may confer antiviral activity during infection. In particular, exosomes secreted by CD8+T cells have been linked with non-cytotoxic suppression of HIV-1 transcription [30]. It was noted that CD8+ T-derived exosomes inhibited HIV-1 transcription in the absence of any viral protein expression [32]. Apolipoprotein B mRNA Editing Enzyme Catalytic Subunit 3G (APOBEC3G or A3G) was observed to be the significant exosomal component responsible for the anti-HIV-1 activity by inhibitory exosomes [79,80]. As anticipated, HIV-1 replication was found to be restrained by A3G at preintegration steps, before the build-up of Vif in those cells that express enzymatically active low-molecular-mass forms of A3G [79,80,81]. It is worth mentioning that some other components of exosomes also inhibit HIV-1 infection, mainly different cytokines, which include interleukins (ILs), interferon-alpha (IFN-α), interferon-beta (IFN-β), and tumor necrosis factor (TNF-α) [82,83,84,85,86]. 

### 4.2. Semen, Vaginal Fluids, Breast Milk, and Other Biological Fluids

Reports suggest that exosomes from healthy individuals inhibit HIV-1 replication by blocking viral RNA reverse transcription. Exosomes secreted from the cells and secreted in biological fluids—semen [87,88,89], vaginal fluids [78], and breast milk [90]—suppress HIV-1 replication. Researchers have reported that HIV-1 replication is blocked by semen and vaginal exosomes at the post-entry stage before integration (e.g., reverse transcription level) [78,89]. CCR5-binding cytokines and CXCR4 ligands in semen inhibit the replication of CCR5- and CXCR4-tropic strains of HIV-1, respectively [91]. Semen clusterin also inhibits the viral HIV-1 infection of dendritic cells by binding to dendritic-cell-specific ICAM-3-grabbing nonintegrin (DC-SIGN). Clusterin competes with HIV for binding sites and thus inhibits the viral entry [92]. However, depletion of clusterin does not restore the HIV binding to DC-SIGN, which shows that multiple factors contribute to the inhibition of HIV entry. The seminal mucin-6 protein was found to be an inhibitor of HIV-1 entry to DCs [93]. However, it is still to be clarified whether mucin-6 or other components of the exosomes isolated from the semen of HIV-1-infected individuals are responsible for HIV-1 inhibition. Therefore, our understanding of the role of semen in HIV-1 infection and its spread remains premature. 

In our studies, we found highly impaired HIV transmission and replication when we incubated the cells with exosomes isolated from vaginal fluid [78]. Specifically, our data demonstrated that vaginal fluid exosomes inhibited HIV by restricting post-entry steps, including reverse transcription and integration [78]. Other studies also reported that EVs derived from vaginal Lactobacillus protect against HIV transmission by blocking its entry into targeted cells [77]. Various studies have suggested that milk exosomes have a strong inhibitory effect against HIV. These exosomes are transferred to the newborn baby via breastfeeding, providing passive antiviral immunity [62]. However, other studies have also shown that exosomes from human breast milk can enhance HIV-1 entry when the virions and exosomes are co-incubated [94,95]. More research is needed to understand the effect of exosomes from human breast milk and other biological fluids. 

Thus far, we know that the content of exosomes varies depending upon their origin, and exosomes derived from the different sources can have a similar effect on HIV pathogenesis (Table 1). However, variation in preparation conditions, such as culture condition, exosome or virion preparation, cell infection status, and exosomal transfer or delivery status, may influence the effect of exosomes in HIV infection [96].

## 5. Wound Healing and HIV Infection

Wound healing (WH), a normal biological process in the human body, begins after a tissue injury to repair and protect the body from further damage due to infection, blood loss, and other complications [98,99]. It is achieved through four highly programmed phases: hemostasis, inflammation, proliferation, and remodeling [27,98,100]. The healing process begins after an injury, starting with hemostasis, which is initiated by vasoconstriction in response to injury to prevent blood deprivation and loss of fluids and electrolytes. Subsequently, nearby platelets infiltrate into the wound site and start to adhere to the exposed collagen. This is then followed by platelet aggregation, where platelets begin to form a plug [100,101,102]. Clotting factors, which are secreted by platelets and the surrounding tissue, prompt fibrin formation. Fibrin is a crucial protein involved in the clotting of blood and is generated upon cleavage of fibrinogen by thrombin [103]. Platelets also facilitate the healing response through the release of pro-inflammatory cytokines and growth factors, including transforming growth factor-beta (TGF)-β and the platelet-derived growth factor (PDGF), which also contribute during the later phases of healing [98,100,101]. TGF-β activates macrophages to release more cytokines such as FGF (fibroblast growth factor), PDGF, tumor necrosis factor-alpha (TNFα), and interleukin-1 (IL-1). (TGF)-β also assists in the expression of collagen and collagenase and improves the chemotaxis of fibroblast and smooth muscle cells [98]. 

After hemostasis, the inflammation phase begins. This phase is essential in the healing process, and a dysfunction in the inflammatory response can lead to poor wound healing [104]. Edema and erythema characterize the initial signs of the inflammatory response at the site of injury. The damaged blood vessel increases its blood flow, which allows leukocytes to infiltrate into the wound area. Resident immune cells, including mast cells, gamma delta T cells, and Langerhans cells, become activated and begin to release cytokines and chemokines. Inflammatory cells allow the release of lysosomal enzymes and reactive oxygen species (ROS) and aid in the removal of cellular debris. Leukocytes are also reported to play a role in the wound healing process during inflammation, and affect many aspects of repair. 

As the inflammatory phase begins to decline, the proliferative phase (rebuilding phase) begins [100,105]. The proliferative phase consists of an overlapping series of events including angiogenesis, collagen remodeling, granulation tissue formation, and epithelialization [104]. During this phase, granulation tissue becomes healthy when it receives a sufficient amount of oxygen and nutrients via the formation of a new network of blood vessels. Normal granulation tissue is red or pink and uneven in texture during the healthy stages of wound healing. The darkening of granulation tissue is a sign of infection, ischemia, or poor perfusion. At the final phase of the proliferative stage, epithelial cells form a new coating at the injury site. Keeping the wound moist and hydrated helps to speed epithelialization. Therefore, an occlusive or semi-occlusive dressing is normally applied within 48 h of injury to maintain optimum tissue humidity. 

Then comes the maturation phase, also called the remodeling phase, in which an attempt to recover the normal tissue structure occurs. During this phase, collagen is remodeled from Type III to Type I, and the wound fully closes. Blood vessels and inflammatory cells gradually start to disappear from the wound area through the process of programmed cell death or apoptosis. In this final stage of the lesion’s healing, collagen fibers become thicker, aligned, placed in parallel, and lie close together to form a cross-link. Cross-linking of collagen is important to make the skin area stronger, and it reduces scar formation. However, the healed wound area remains weaker compared to the uninjured normal skin, even after the formation of cross-linked collagen. Generally, remodeling starts 21 days after an injury and continues up to one year or more. 

To heal a wound successfully, all four phases (Table 2), hemostasis, inflammation, proliferation, and remodeling, must occur in the proper sequence and also within a certain time frame. Failure to progress in any of the four stages of wound healing can lead to chronic wounds. Many factors can affect wound healing by interfering in any of the four phases, thus causing improper or impaired tissue repair. Critical factors that can influence and impair the healing process have gained attention recently—factors like age, stress, sex hormones, diabetes, medications, weight, nutrition, alcohol consumption, smoking oxygenation, and infection are known to influence the wound-healing process. These factors can be classified as local factors and systemic factors. Local factors influence the characteristics of the wound directly, while systemic factors relate to the overall health or disease state of the individual that affects the ability to heal the lesion [100]. Infection, oxygenation, and mechanical stress are among the factors that are considered to influence wound healing directly while age, sex, hormones, obesity, diabetes, medication, alcoholism, nutrition, and immunodeficiency are the systemic factors that act through local effects to impair wound healing. One or more factors may play a role in any of the four wound-healing phases and influence the overall outcome of the healing process [100].

In many cases, infection with pathogens remains the main hindrance to wound healing. When tissue is injured, macrophages and neutrophils provide the first line of defense against invading pathogens. They are part of the innate response. They display receptors on their surface that recognize common characteristics of various pathogens, as do dendritic cells (DCs) [106]. Macrophages, neutrophils, and DCs are stimulated by the binding of a microorganism substance or antigen to their surface receptor [106]. Once stimulated, these cells undergo phagocytosis. If the innate response is unsuccessful in destroying the invading pathogen, the adaptive response plays its part. DCs mature into an antigen-presenting cell (APC) and migrate to the peripheral lymphoid organs to stimulate T lymphocytes by presenting the pathogen antigen [106]. T lymphocytes become activated upon binding to the antigen, which causes clonal proliferation of specific T cells. A large number of T cells migrate to the site of infection to kill infected cells and produce cytokines to stimulate B cells. Upon stimulation, B cells differentiate into antibody-producing plasma cells that flag pathogens for destruction [106,107,108]. Wound infection can cause complications in wound healing, particularly if the immune system is not able to clear the infection from the site of injury. This may lead to persistent inflammation and a failure to heal [100,109]. In short, infection is a significant complication for wound healing, and the immune response is crucial to the resolution of this complication. 

HIV patients with acquired immunodeficiency syndrome (AIDS) have an impaired immune system and poor immunity. They are known to be highly susceptible to wound infection [7,8,110]. Studies have shown that the decline of CD4+ cells in AIDS patients correlates with impaired wound healing [8] and prolonged inflammation [48]. Other studies have shown that the healing of wounds and severity of wound complication does not correlate with the CD4 count [111]. However, details concerning the rate of wound healing and its correlation with CD4+ cell counts and viral load are still premature [112]. Reports have also suggested that HIV patients with AIDS have a significantly higher risk of wound infection and other wound-healing-related complications in comparison to HIV patients without AIDS [8]. ART-naïve HIV-infected individuals with CD4 counts of less than 350 cells/µL experience a slower rate of healing than those with CD4 counts above 350 cells/µL, according to published research [112,113]. However, HIV status alone is not associated with a significantly longer healing period [114]. Thus, generally, it is noted that HIV-infected patients without AIDS are at relatively lower risk of wound-healing complications [8]. To date, no study indicates that HIV infection itself is an independent risk factor for complicated wound management. Open research areas include investigating the role of HIV status in the formation of chronic wounds. Nevertheless, a large number of HIV patients without AIDS present with other metabolic syndromes, such as diabetes [115], which puts them under an increased risk of developing wound-healing complications [116]. Patients with Type 2 diabetes have impaired immunity, which contributes to wound-healing impairments [117,118]. Moreover, infection with HIV enhances the chances of other coinfections to the host, especially fungal infections on the skin. These fungal infections can spread through the wound, resulting in impaired healing and increased morbidity, hospitalization, sepsis, reoperation, and even death [110].

## 6. Effect of EVs in the Process of Wound Healing

Cells of various types are involved in wound healing. These cells release EVs into extracellular environments. EVs derived from non-immune and immune cells also play a significant role in the regulation of the immune system [17]. Content transfer through cell-to-cell communication mechanism enables EVs to regulate cell proliferation and growth factor expression in the absence of cell contact [119]. Studies have shown that EVs regulate ECM and are responsible for elevating the healing process along with reducing scar area in the rat model [120]. EVs are associated with ECM synthesis through the increased release of the ECM protein elastin. The role of EVs in many wound healing steps, including coagulation [121,122,123], cell proliferation [20,124,125,126,127], cell migration [20,22,124,125,128,129,130,131], and remodeling [20,124,132,133,134], have been documented in many studies. 

### 6.1. Role of EVs in Coagulation

**Figure 3:**
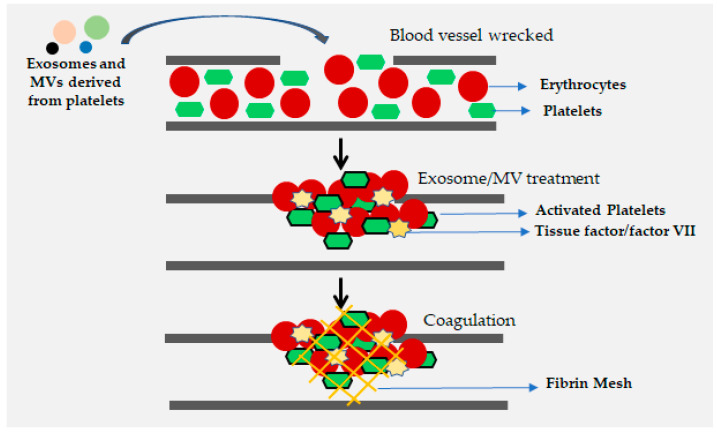
Enhanced coagulation via exosomes and MVs derived from platelets through tissue factors.

Blood coagulation is known to be initiated through the tissue factor (TF), a protein that functions in thrombin formation by converting zymogen FX to its active form FXa [122]. Microvesicles (MVs) and exosomes that carry tissue factor (TF) can be derived from platelets, monocytes/macrophages, and saliva. EVs, including microvesicles and exosomes derived from human saliva platelets, and monocytes/macrophages are reported to influence the process of coagulation. The tissue factors present in saliva, along with coagulation factor VII, have been shown to promote the coagulation process (Figure 3) and reduce the clotting time [122]. Therefore, EVs may help to minimize blood loss and protect the body from pathogen invasion [27,135]. Additionally, the phosphatydilserine-enriched membranes of MVs derived from platelets, including exosomes, serve as a surface for attachment of clotting factors that aid in coagulation [128]. 

### 6.2. Role of EVs in Inflammatory and Immune Response

Various immune cells, including mast cells [136], macrophages [137,138], dendritic cells [139], T cells [140,141], and B cells [142], secrete EVs. Immune- and non-immune-cell-derived EVs, particularly exosomes, play a significant role in the regulation of the inflammatory response [143,144]. EVs are involved in the inflammatory response via intercellular communication between cells and could be involved in long-term immune memory. Neutrophil-derived EVs can also exert anti-inflammatory effects. EVs secreted from neutrophils induce downregulation of the transcription of pro-inflammatory cytokines and allow the release of TGF-β1 from macrophages [145,146]. EVs derived from platelets have an anti-inflammatory effect. They reduce the production of interferon γ (IFNγ), TNFα, and IL-6 secretion from T cells [147]. RBC-derived EVs can increase the phagocytic activity of neutrophils in humans by triggering an increase in CD11b. EVs also regulate the immune system’s transport of inflammatory mediators and receptors. EVs derived from monocytes have pro-inflammatory effects through their interactions with various cells, including endothelial cells, other monocytes, fibroblasts, and smooth muscle cells. EVs play a crucial role in the release of interleukin (IL)-1β [148], a pro-inflammatory cytokine that is essential for host-defense responses [49]. Furthermore, EVs derived from monocytes carried by interleukin-1 were found to activate endothelial cells and stimulate the generation of IL-1β from monocytes [149]. Depending on the source of secretion, the activities of exosomes against pathogens vary. Exosomes derived from mature DCs aid in T-cell and NK-cell activation [150]. Exosomes secreted from T cells can either activate or suppress the immune system, depending on the activation status and tissue microenvironment of the T cell and other factors [32,151,152]. Activated CD3+ T cells communicate with resting T cells through exosomes [32]. Exosomes released from CD4+ T cells are capable of delivering different signals, such as antigen-specific signals, atherogenic signals, and co-stimulatory signals [153]. Exosomes derived from various immune cells serve an essential purpose in wound healing and repair [132]. 

### 6.3. Role of EVs in the Proliferation Phase

EVs, including exosomes, are reported to play a significant role in mediating different parts in the proliferative phase of wound healing. Shabbir et al. have shown that exosomes secreted from mesenchymal stem cells (MSCs) activate several signaling pathways [20] that modulate wound healing. Exosomes promote cell proliferation by enhancing the expression levels of hepatocyte growth factor (HGF), insulin-like growth factor-1 (IGF1), nerve growth factor (NGF), stromal-derived growth factor-1 (SDF1); increase re-epithelialization; reduce scar widths; promote the maturity of collagen and create new vessels; support wound-site maturation vessels; and activate Akt, Erk, and Stat3 signaling [20,124]. Li et al. (2016) found that exosomes derived from endothelial progenitor cells (EPCs) facilitate wound healing by positively modulating vascular endothelial cell function [130]. Zhang et al. (2015) reported on the potential of exosomes derived from human-induced pluripotent stem-cell-derived mesenchymal stem cells (hiPSC-MSCs) for treating cutaneous wounds for the first time. The team suggested that hiPSC-MSC-Exos can facilitate cutaneous wound healing by promoting collagen synthesis and angiogenesis [124]. Moreover, studies have also shown that exosomes secreted from platelet-rich plasma (PRP) promote the re-epithelization of chronic cutaneous wounds [129]. Meanwhile, a study by Cheng et al. (2018) showed that exosomes secreted from human keratinocytes (HKC) could not promote cell proliferation [125]. Their content of extracellular hsp90α overrode TGF-β inhibition to promote dermal cell migration. 

It has also been hypothesized that exosomes derived from adipose-derived stem cells (ASCs) promote the migration of fibroblasts toward the site of injury through internalization [154]. A study in vitro found that exosomes can enter fibroblasts’ cytoplasm and secrete an active substance into the cells that influences fibroblast migration, proliferation, and collagen secretion [155]. EVs derived from human adipose-derived MSCs accelerate the migration and proliferation of dermal fibroblasts and keratinocytes [156]. These EVs also activate the AKT pathway [156]. Ferreira et al. (2017) concluded that EVs are a promising tool for wound healing [156]. 

EVs derived from lymphocytes have also been documented as having pro- or anti-angiogenic effects. Research has shown that activation and apoptosis of lymphocytes releases MVs with pro-angiogenic properties, whereas microparticles released from apoptotic lymphocytes inhibit angiogenesis [157,158,159]. EVs with abundant micro-RNA (miR-214 and miR-126) appear to be capable of inducing pro-angiogenic signaling in adjacent epithelial cells [160]. However, EVs derived from platelets were discovered to have anti-angiogenic properties, inhibiting angiogenesis by altering subunits of NADH oxidase and increasing oxidative stress [161]. Taken together, EVs seem to have a positive effect on the proliferation phase and stimulate proliferation, migration, and collagen secretion. EVs can also have both pro- and anti-angiogenic effects, the overall balance of which likely influences angiogenesis and wound healing. However, an in-depth understanding of the role of EVs remains to be elucidated.

### 6.4. Role of EVs in Remodeling

EVs play a crucial role in regulating extracellular matrix (ECM) remodeling, the last phase of wound healing, particularly by promoting the production of ECM proteins such as elastin and collagen [27,129]. Studies have found that mesenchymal stem cell (MSC)-derived exosomes promote collagen I and III produced during earlier stages of wound healing [27,129]. Furthermore, studies have also found that exosomes reduce scar formation by preventing collagen production during the late stage of wound healing [132]. EVs may aid in the formation and function of the ECM, since they are linked with the collagen network of the ECM. In addition to ECM remodeling, research has found that EVs notably improved the healing rate and decrease scar diameters in a rat model [27]. MVs derived from endothelial cells also contain matrix metalloproteinases, which are involved in the tissue remodeling phase [27,162,163]. Additionally, during wound healing, embryonic MSC- and endothelial-cell-derived exosomes prompt endothelial cell generation and migration, which aid in angiogenesis. A previous study has found that exosomes derived from mesenchymal stem cells (hiPSC-MSC-Exos) play a significant role in wound healing and repair. HiPSC-MSC-Exos were found to improve cutaneous wound healing, collagen synthesis, and angiogenesis at the wound site in a full-thickness skin-defect rat model [131].

## 7. Future Perspectives on EVs in HIV Infection and Wound Healing

EVs are considered a “fingerprint” of cells as they reflect the condition of the cells [164]. EVs (particularly exosomes) could be used as biomarkers to detect cellular abnormalities, even infection to the cells. Exosomes are considered effective and sturdy biomarkers because of their stability, sensitivity, and specificity [165]. Welker et al. (2012) suggested that exosomal CD81 may be a potential marker for hepatitis C diagnosis and treatment response, as the level of serum exosomal CD81 is elevated in patients during chronic hepatitis and severe fibrosis [166]. Exosomal EGFRvIII may also provide diagnostic information for glioblastoma [167]. It is reported that exosome from the serum of brain tumor patients has an elevated level of EGFR, EGFRvIII, and TGF-beta; therefore, it could be used as a biomarker [168]. Further, studies have shown that tau phosphorylated at Thr-181 is present at elevated levels in exosomes isolated from cerebrospinal fluid specimens of Alzheimer’s disease (AD) patients [169]. 

Likewise, exosomes could also be used as biomarkers for HIV-1 infection. The presence of HIV-1 proteins and RNA in the exosomes from HIV-1-infected patients accentuates the potential use of circulating exosomes as a biomarker for HIV-1. During HIV infection, exosome-associated immune and oxidative stress markers may be used as indicators of HIV-1 disease progression. Furthermore, an abundance of plasma exosomes and the size of exosomes correlate inversely with CD4 counts and correlate positively with CD8T cell counts, thus indicating HIV disease progression [63]. On the other hand, as the EVs, particularly exosomes, derived from semen [87,88,89], vaginal fluids [78], and breast milk [90] have been identified as inhibitory to HIV, these exosomes could be used as a potential therapy against HIV infection. These EVs have protective properties that can restrain vertical and horizontal viral transmission. They could be isolated and used as a natural carrier of anti-HIV-1 molecules, thereby preventing HIV infection and its progression. Even though the beneficial roles of these exosomes are known, the mediators are still yet to be identified. Indeed, it will be important to characterize the exact component(s) and the mechanism responsible. The delivery of antiviral molecules and/or therapeutic vaccines utilizing EV-based delivery systems could represent a major improvement in drug development. Certainly, EVs are much more likely to have low immunogenicity compared to liposome- and lentiviral-based delivery systems. The ease of engineering these exosomes and the non-synthetic nature of these delivery systems offer advantages for disease targeting.

The roles of exosomes in the field of wound repair and cutaneous regeneration have gained a lot of attention over the last few decades. Therapies based on exosomes derived from mesenchymal stem cells (MSCs) have emerged as a promising technique for their ability to promote wound healing and minimize scarring [170]. Despite having issues pertaining to the separation of a highly pure and uniform exosomal fraction [171], EV-based therapies have many advantages, including being easy to prepare, store, and transport, easy to dose, and easy to administer. Moreover, they have high therapeutic efficiency with minimum risk of immune rejection and tumorigenesis. These advantages make them useful in regenerative medicine without the limitations of cellular therapy. Consequently, MSC-exosomes have potential for cutaneous regeneration and could effectively replace whole-MSC-based therapy. Since the exosomes have regenerative attributes like stem cells and may avert undesired effects associated with stem-cell transplantation, exosomes can be used effectively for direct treatment. Exosomes cause angiogenesis [172], promote proliferation, skin-cell migration, and wound closure, and enhance the healing process in animal models [131,173] when administered locally as an injection. This suggests that exosomes offer a promising therapeutic approach for wound healing.

## 8. Conclusions

EVs are rapidly evolving and expanding topics in the field of biology, affecting almost all biomedical disciplines including HIV/AIDS and wound healing. However, many questions about EVs remain, as do many challenges to their use. A major hurdle in understanding the specific functions of EVs is the inability to separate and classify the complex population of vesicles into subclasses of particular sizes, compositions, and biogenetic pathways. Since various factors, such as the cellular origins, recipient cells, and the intracellular signaling, influence the role of EVs in HIV infection, the preparation and testing conditions play a crucial part. The role of EVs appears to vary considerably during HIV infection. Due to their ability to modulate the HIV lifecycle, it is expected that a certain population of EVs could be developed as a biomarker for HIV infection, besides their use as potential therapeutics. Because EVs also play crucial roles in overlapping phases of wound healing, including coagulation, inflammation, cell proliferation, cell migration, angiogenesis collagen production, and ECM remodeling, they could also be a potential tool in wound-healing treatments. Although significant advances have been made in the role of EVs, a more in-depth understanding is still required, in particular in intercellular communication, immune modulation, and immune surveillance. Compared to other fields, the role of EVs in HIV infection and wound healing remains to be explored. Therefore, more research should be anticipated at the in vivo level to reveal the potential of EVs in the development of anti-HIV therapy. Understanding the effect of weak immunity on EV function in wound healing in HIV-infected individuals will be of great significance in understanding the therapeutic potential of EVs in the wound healing process, especially of HAART-taking longer-living HIV patients. 

## Figures and Tables

**Table 1:** List of exosomes shown to modulate HIV infection/transmission/disease progression.

Impact on HIV-1 Progression	Exosome Source	Reference
Enhance	Exosomes from HIV-infected plasma (blood) Exosomes from HIV-infected serum (blood)	[63,64,65,66,67,68,69,70,71,72,73,74,75,76,97]
Inhibit	Exosomes from uninfected human semen Exosomes from uninfected human vaginal fluid	[87,88,89] [78]
Enhance/reduce	Exosomes from uninfected human breast milk	[62,94,95]
Not significant	Exosomes from uninfected plasma (blood)	[96]

**Table 2:** The process of normal wound healing.

Phases		Events
**1st phase**	Hemostasis	Vasoconstriction, platelet infiltration and aggregation into the wound site, and fibrin formation
**2nd phase**	Inflammation	Increased blood flow (neutrophil, monocyte, and leucocyte infiltration) and activation of resident immune cells to release cytokines and chemokines
**3rd phase**	Proliferation	Angiogenesis, collagen remodeling, granulation tissue formation, and epithelialization
**4th phase**	Remodeling	Collagen remodeling, vascular maturation, and regression
